# Some Probationers Articulate

**Published:** 1918-10-19

**Authors:** 


THE EDITOR'S LETTER-BOX—(*continued*).

### Some Probationers Articulate.

*To the Editor of* The Hospital.

Sir,—In reference to the statements which appeared in The Hospital on October 11, and which we think give the public a somewhat erroneous idea of the conditions which exist, would you kindly find room to insert the reply from some probationers of the London Hospital?

The article strikes us as having been written more out of personal spite than with a view to improving the nursing profession.

We write to make it quite clear that the correspondent is not voicing the feelings of the present London Hospital probationers. It is made absolutely clear to all candidates what is expected of them when they enter the London Hospital, before they sign any agreement whatever. Surely after seven weeks at the preliminary training school, and a month's trial in the hospital wards, they should have sufficient idea of the daily life of a probationer, and if not satisfied are quite free to leave.

Except for a short period of night duty, probationers' classes are not taken from their off-duty time, but extra time is allowed for them.

With regard to sweeping and the routine work, these duties are indirectly as necessary to the welfare of the patient as any personal attention. Such duties are necessarily part of a nurse's training, especially in her first year, but only a small part compared with the practical, medical, and surgical nursing that our sisters and staff nurses do their utmost to teach us.

Regarding meals on night duty, our night sisters are much too severe on this point to ever allow a nurse to miss a meal; but surely any woman training as a nurse should be willing to sacrifice a part of her meal-time, if necessary, to make a suffering fellow-creature more comfortable.

We feel that the remarks of the correspondent are most self-centred and her standard very low; certainly not that aimed at by London Hospital probationers. We look upon nursing as a calling, and not to be classed with ordinary professions. Moreover, we are not in terror from day to day of being sent away from hospital; we know that this will only happen if we fail to come up to the standard of the London Hospital, and it is that standard of which we are all so proud.

We are perfectly happy and satisfied that we are being treated fairly, and if we have any complaints to make we know that those in authority will regard them with consideration and justice. We feel very strongly that our matron and chairman have always worked, and are still working, for the welfare and happiness of the nursing staff, and we need no other champion.

We, the writers, only ask you, Sir, to publish this because we are assured that it expresses the popular feeling amongst us.

(*Signed*) London Hospital Probationers.

Nurses' Home, London Hospital, E. 1.

### The Shortage of M.A.B. Nurses.

*To the Editor of* The Hospital.

